# Consumer opinion on the use of machine learning in healthcare settings: A qualitative systematic review

**DOI:** 10.1177/20552076241288631

**Published:** 2025-01-06

**Authors:** Jacqueline H Stephens, Celine Northcott, Brianna F Poirier, Trent Lewis

**Affiliations:** 1 568638 Flinders Health and Medical Research Institute, College of Medicine and Public Health, 1065 Flinders University, Adelaide, Australia; 2 367695 South Australian Health and Medical Research Institute, Adelaide, Australia; 3 The University of Adelaide, Adelaide, Australia; 4 College of Science and Engineering, 1065 Flinders University, Adelaide, Australia

**Keywords:** Machine learning, artificial intelligence, consumers, systematic review, qualitative research

## Abstract

**Introduction:**

Given the increasing number of artificial intelligence and machine learning (AI/ML) tools in healthcare, we aimed to gain an understanding of consumer perspectives on the use of AI/ML tools for healthcare diagnostics.

**Methods:**

We conducted a qualitative systematic review, following established standardized methods, of the existing literature indexed in the following databases up to 4 April 2022: OVID MEDLINE, OVID EMBASE, Scopus and Web of Science.

**Results:**

Fourteen studies were identified as appropriate for inclusion in the meta-synthesis and systematic review. Most studies (*n* = 12) were conducted in high-income countries, with data extracted from both mixed methods (42.9%) and qualitative (57.1%) studies. The meta-synthesis identified four overarching themes across the included studies: (1) Trust, fear, and uncertainty; (2) Data privacy and ML governance; (3) Impact on healthcare delivery and access; and (4) Consumers want to be engaged.

**Conclusion:**

The current evidence demonstrates consumers’ understandings of AI/ML for medical diagnosis are complex. Consumers express a complex combination of both hesitancy and support towards AI/ML in healthcare diagnosis. Importantly, their views of the use of AI/ML in medical diagnosis are influenced by the perceived trustworthiness of their healthcare providers who use these AI/ML tools. Consumers recognize the potential for AI/ML tools to improve diagnostic accuracy, efficiency and access, and express a strong interest to be engaged in the development and implementation process of AI/ML into routine healthcare.

## Introduction

Artificial intelligence and machine learning (AI/ML) technologies are rapidly transforming the healthcare industry. Tools using these technologies are being developed to improve the accuracy and timeliness of medical diagnosis for a wide range of health conditions,^1–5^ as well as to analyse existing patient medical and pathology data to predict future health diagnoses,^6,7^ and support doctors to develop personalized treatment plans.^8,9^ While the growth of this industry has been rapid – clearly evidenced by the exponential growth in the peer-reviewed literature – there is a range of potential risks in the development and implementation of these tools. These risks include patient data privacy,^10^ cybersecurity vulnerabilities,^11^ the development of biased tools^12,13^ and a lack of interpretability of the AI/ML output.^1,7,14,15^ Given these potential risks, and the inevitable impact of AI/ML tools on the population, it is critical to consider the opinions of consumers on the use of these tools in healthcare.

Consumer opinion is essential for the successful adoption and integration of any new technology, and this is particularly heightened in the context of AI/ML diagnostic tools for use in healthcare.^16,17^ In fact, research shows consumers are accepting of, and engage with, a range of healthcare technologies, such as chatbots and wearable activity trackers. Furthermore, these technologies are positively influencing consumers’ health, with chatbots proving effective in positively altering lifestyle behaviours^18,19^ and wearable activity trackers increasing physical activity.^20^ The increasing acceptance and use of these medical devices by consumers is evident in their ever-increasing purchase and ownership. The most recent data suggest up to a third of the population own and use a wearable device, such as a smartwatch,^21,22^ with the main uses identified as monitoring athletic performance, sleep patterns and heart health.^22^ The widespread purchase and use of these tools can be taken as a proxy for understanding consumer acceptance of AI/ML in healthcare, and may indicate consumers are keen to embrace technology to self-monitor their own health. However, given consumer perspectives of third-party use of health system data is complex and still not well understood,^23,24^ it is unsurprising there is a scarcity of data on consumer perspectives of data use by the companies producing these medical devices and AI/ML tools. However, consumer opinion will most certainly influence the acceptability of these AI/ML tools, and influence whether healthcare providers choose to adopt these and integrate them into their healthcare delivery.

Recent advances in AI/ML tools and their release to the consumer market have brought the potential use and application of these to the forefront of consumer consciousness. These tools, such as generative AI (for example, ChatGPT), are developing faster than consumers and governance structures can adapt. Currently, there is limited legislation governing the use of AI/ML in healthcare. As such, there is also a lack of legislation protecting how patients’ data are used in the development of these tools. In Australia, key medical organizations have produced guidelines providing direction for the ethical and responsible use of AI/ML in healthcare;^25–27^ however, a global understanding of the legal implications of AI/ML for healthcare is unclear.^28^ Importantly, understanding the concerns of consumers about the use of their data in the development of AI/ML tools for healthcare is an important step in ensuring these are developed and used in a way that is acceptable to the population. The aim of this systematic review is to examine and collate the existing literature on the opinions of consumers regarding the use of AI/ML in healthcare diagnostic tools.

## Materials and methods

This systematic review has been registered with the International Prospective Register of Systematic Reviews (PROSPERO) (CRD42022323061). This qualitative systematic review has been reported in alignment with both the Enhancing Transparency in Reporting the Synthesis of Qualitative Research (ENTREQ) statement^29^ and the Preferred Reporting Items for Systematic Reviews and Meta-Analyses (PRISMA) guidelines (Supplemental File 1).^30^ We used an online tool to prepare the PRISMA flowchart.^31^

### Identifying studies for inclusion

The researchers employed a pre-established search strategy,^32^ which utilized key terms (and their variants) related to the population of interest, the study design, and the phenomenon of interest. The search was tailored for each of the following databases: OVID MEDLINE, OVID EMBASE, Scopus and Web of Science (Supplemental File 2). Articles published from database inception until 4 April 2022 identified through the systematic search were exported into Covidence, a web-based collaboration software platform used to facilitate the systematic review process.^33^ A search update was conducted on 31 July 2024. Dual screening of article titles and abstracts was conducted independently by a combination of three reviewers (BP, JS and CN) to determine eligibility. Two reviewers (JS and CN) independently screened articles’ full text against predetermined inclusion criteria (Table 1). Any disagreements arising during the review process were resolved by consensus discussions. While efforts were made to minimize the impact of publication bias, the review team recognizes limiting inclusion criteria to the English language could have resulted in a loss of data. Further, the inclusion of grey literature may have provided more findings for inclusion in this review.

**Table 1.** Inclusion criteria for study selection.

Inclusion criteria
The article reported original research.
The study design was qualitative or mixed methods (with clear qualitative illustrations).
The study included consumer opinion of the use of machine learning in healthcare settings.
Findings contained qualitative illustrations, e.g., participant quotes.
The article was available in the English language.
The article was published prior to 4 April 2022.
The article was available as full text in downloadable or hardcopy form.

### Critical appraisal

Several validated tools exist for the appraisal of qualitative studies. For comprehensive critical appraisal, we utilised two tools: the Critical Appraisal Skills Programme (CASP) Qualitative Studies Checklist^34^ and the Joanna Briggs Institute (JBI) checklist for qualitative research.^35^ While the CASP checklist is useful to assess the strengths and limitations of the included qualitative research,^34^ in comparison to other appraisal tools it has been found to be less sensitive for evaluative and theoretical validity.^36^ In comparison, the JBI appraisal tool has been identified to have greater sensitivity when assessing validity and congruity.^36^ Therefore, JBI was used to evaluate agreement between research philosophies, findings and methodologies as well as researcher positionality.^35^ All included studies were appraised using both tools by two reviewers (JS and CN), which provided a more conclusive and complementary assessment of the included studies. Interrater reliability was evaluated to assess the consistency in the appraisal decisions made by the two independent reviewers. Statistical measurement of interrater reliability through Cohen's kappa coefficients were calculated and reported, with a kappa value above 0.61 considered good agreement.^37^

### Data extraction and synthesis

Data extraction occurred in two phases. The first phase, conducted within the Covidence platform, involved the extraction of study characteristics, including study location, aims and participant characteristics. Two researchers (JS and CN) independently performed data extraction, resolving discrepancies during an in-person roundtable discussion. In the second phase, PDFs of included studies were imported into NViVO software (version 20, QSR International, Australia). Within NViVO, a comprehensive process of identifying the studies’ qualitative data and associated themes was undertaken independently by two researchers (JS and CN). While both the original participant quotes and the authors’ interpretation of the findings were identified for inclusion in the meta-synthesis, these were synthesized separately. The synthesis of extracted data included reviewing all findings, labelling of common phrases or concepts and generation of codes. The codes arising from the two data types were then further synthesized to determine commonalities. These codes were further synthesized to develop categories encompassing common concepts. Categories were then collated during the final phase into overarching synthesized themes.^38^ Using Microsoft Bing CoPilot^TM^ the key themes were represented visually and are presented in the Supplemental File.

### Role of the funding source

This study was supported by funding from the Flinders Foundation and Flinders University. The funding sources had no input into the study design; collection, analysis or interpretation of data; in the writing of the report; nor in the decision to submit the paper for publication.

## Results

### Sources of evidence

**Figure 1.**
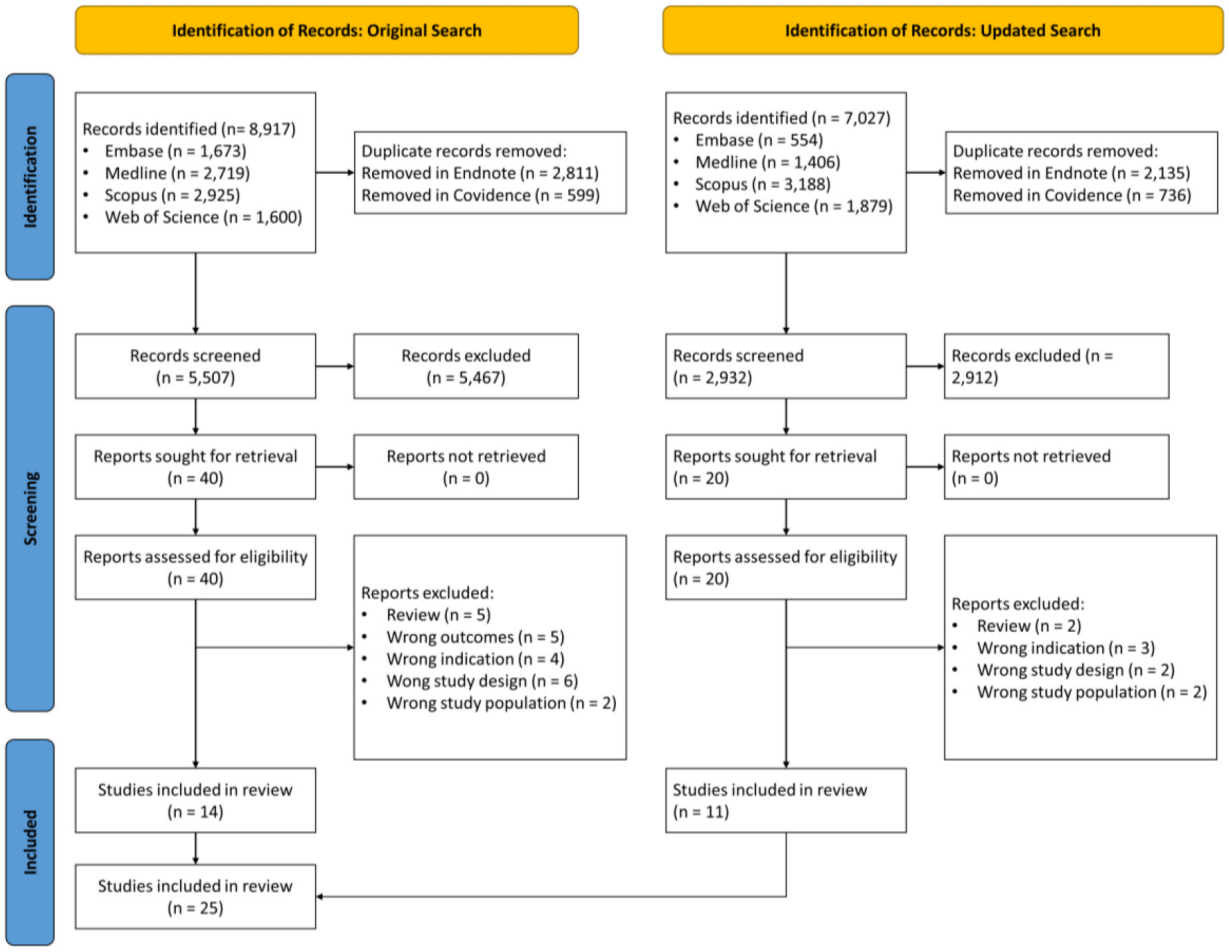
PRISMA flowchart for the selection of included studies.

The original search strategy identified 8917 papers for review (Figure 1). Following removal of 3410 duplicates, 5507 unique papers were dual-screened with 40 selected for full-text review. Of these, 14 studies were identified as appropriate for inclusion. Just prior to publication, an updated search and screening of new literature identified an additional nine studies for inclusion. As such, a total of 23 papers are included in this systematic review. According to the JBI Levels of Evidence, we report a systematic review of descriptive studies (Level 4a).^39^

### Characteristics of sources of evidence

The characteristics of the 23 included studies are summarized in Table 2. The details about each paper are presented in Supplemental File 3. Most of the studies (*n* = 20, 87.0%) were conducted in high-income countries, as defined by the World Bank, with the largest number of studies conducted in the United States (*n* = 8, 34.8%). Data were reported by both mixed methods (26.1%) and qualitative (73.9%) studies using a range of analytical approaches, including thematic analysis (*n* = 12, 52.2%) and grounded theory (*n* = 4, 17.4%). Most of the studies used either semi-structured interviews (*n* = 11, 47.8%) or focus groups (n = 8, 34.8%) to generate the qualitative data. Consumer populations involved in the studies were mainly people seeking oncology treatment (*n* = 5, 21.7%), cancer screening (*n* = 4, 17.4%), or mental healthcare (n = 4, 17.4%).

**Table 2.** Summarized characteristics of included studies (*n* = 23).

**Characteristic**	***n***	**%**
**Country**		
North America	9	39.1%
Europe and Central Asia	8	34.8%
East Asia and Pacific	3	13.0%
Sub-Saharan Africa	2	8.7%
South Asia	1	4.3%
**LMHIC status**		
High	20	87.0%
Lower middle	3	13.0%
**Study design**		
Qualitative Study	17	73.9%
Mixed Methods Study	6	26.1%
**Study population**		
Target population, adults	19	82.6%
Caregivers of target population	2	8.7%
General population, adults	1	4.3%
Not stated	1	4.3%
**Health condition of interest**		
Oncology	5	21.7%
Cancer screening	4	17.4%
General	4	17.4%
Mental health	4	17.4%
Chronic disease	2	8.7%
Radiology	2	8.7%
Respiratory	1	4.3%
Surgical outcomes	1	4.3%
**Data collection method**		
Semi-structured interviews	11	47.8%
Focus group	8	34.8%
Survey w/ free text responses	2	8.7%
Democratic deliberation/Workshop	2	8.7%
**Data analysis approach**		
Thematic analysis	12	52.2%
Content analysis	5	21.7%
Grounded theory	4	17.4%
Descriptive	2	8.7%

LMHIC: low middle high income country status.

### Quality appraisal

Using the CASP tool, we assessed eight of the papers as high quality, that is, the papers fulfilled all 10 CASP criteria. Two papers were assessed as low quality as they only met 3 of the 10 CASP criteria. The remaining 13 papers were of varying quality as presented in Table 3. There was fair agreement in interrater CASP assessment (Cohen's kappa = 0.211). This is most likely due to differences in the experience of the two researchers, with the more senior researcher more critical of the papers.

**Table 3.**
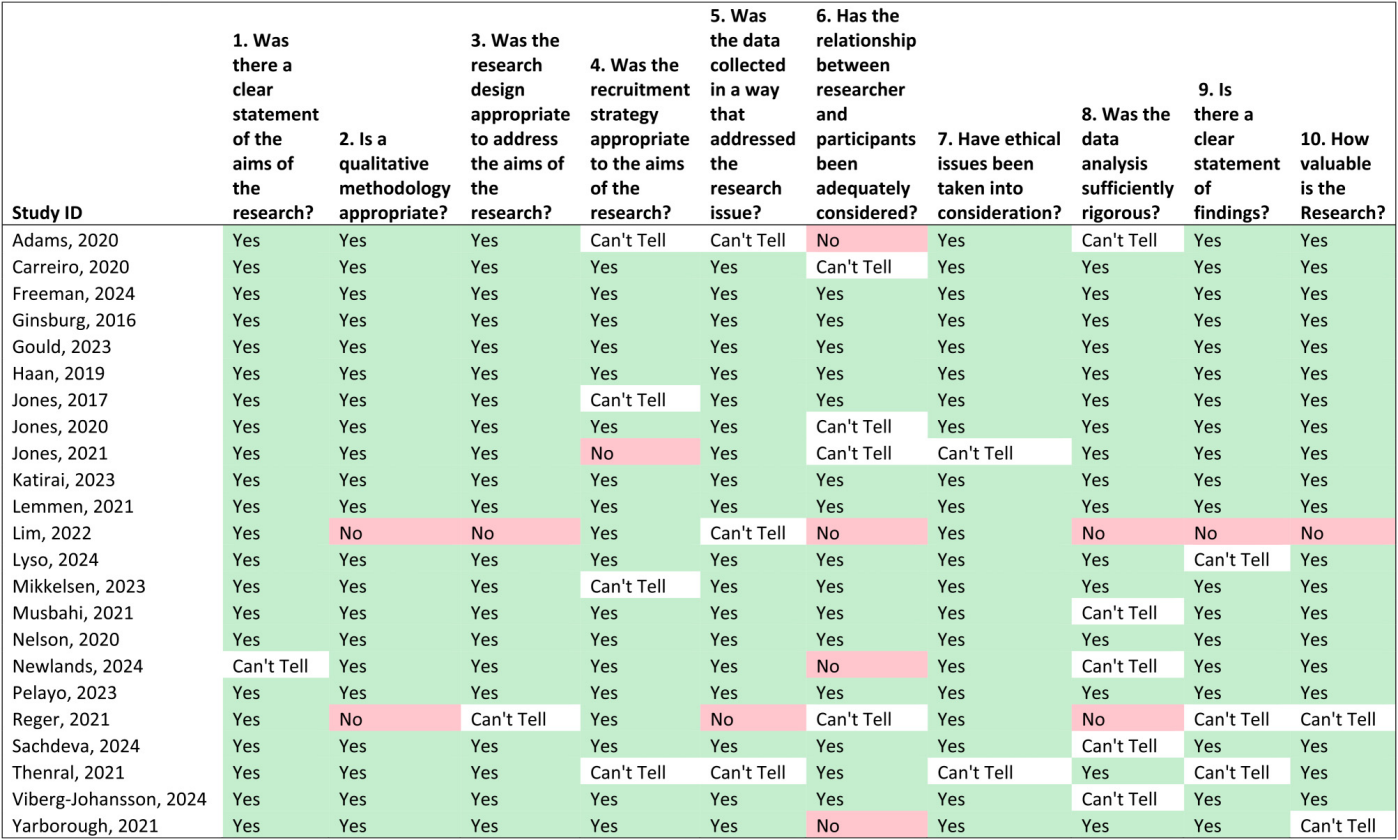
Critical appraisal of included studies using the critical appraisal skills programme (CASP) qualitative studies checklist (*n* = 23).

In contrast, using the JBI critical appraisal tool, we found 12 papers met at least 8 of the 10 criteria for reporting qualitative research, with three meeting all 10 criteria. Two papers were assessed as poor quality as they only met 1 of the 10 criteria (Table 4). All the papers reported ethical or institutional review board outcomes; however, only nine (39.1%) papers included details of the researchers’ cultural/theoretical positionality in relation to the participants. The interrater agreement between the two reviewers using the JBI critical appraisal tool was moderate (Cohen's kappa = 0.549).

**Table 4.**
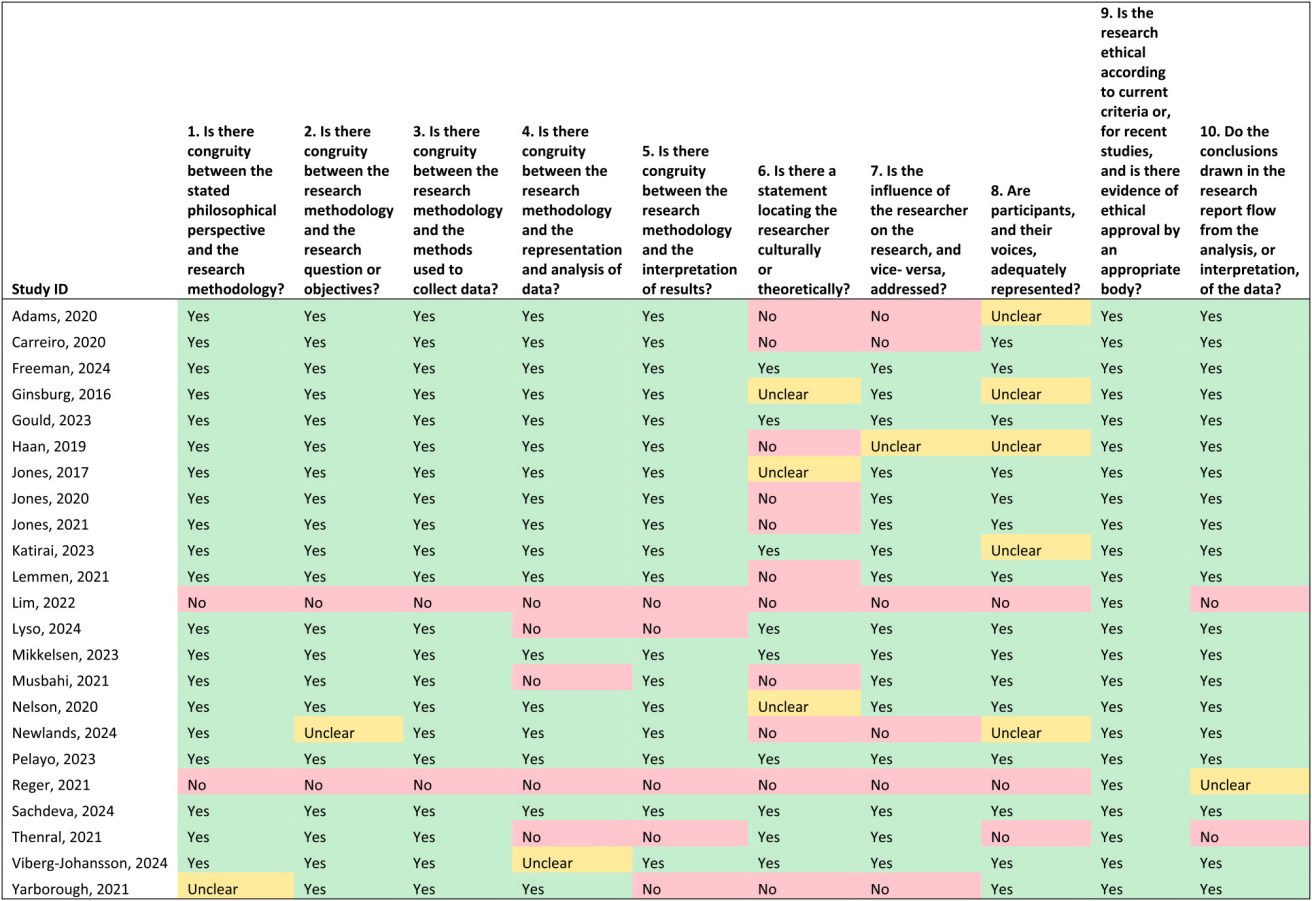
Critical appraisal of included studies using the JBI qualitative studies checklist (*n* = 23).

### Synthesis of evidence

During the meta-synthesis, four overarching themes were identified: (1) Trust, fear and uncertainty; (2) Data privacy and ML governance; (3) Impact on healthcare delivery and access; and (4) Consumers want to be engaged. These themes each incorporated various subthemes, and these will be explored in detail with selected quotations from the original source to provide illustration. A series of AI-generated illustrations conceptualize the four themes to convey how the AI/ML industry has a role as data custodians (Supplemental File).

#### Theme 1: Fear, uncertainty and distrust

Within the literature, consumers expressed fear, uncertainty and distrust about the use of AI/ML for healthcare diagnostics.^40–43,45–47,50,55,56,58,60^ These three distinct but intertwined subthemes are discussed below.

#### Fear

Consumers’ fear of AI/ML was caused by a lack of knowledge about how AI/ML could be used to support healthcare diagnostics – ‘fear of the unknown’.^40,56^ Fear has also been expressed about the potential for future uncontrollable development of AI/ML,^50^ as well as the perception AI/ML could lead to the gamification of health.^40,55^ Consumers were also concerned using AI/ML in healthcare caused, or could create, anxiety.^41,43,50,55,58^ There was evidence consumers mistook the use of AI/ML as an indication of disease severity.^43,55^ Consumers had expressed the use of AI/ML devices to record health metrics could be ‘uncomfortable’ and cause ‘negative experiences’.^41,43^ There was also the proposition AI/ML for disease risk profiling would create an ‘emotional burden’ with a concern consumers ‘believed that knowing one’s prognoses and the practice of predicting disease risks would result in a “self-fulﬁlling prophecy”’.^50^

#### Uncertainty

The uncertainty about the use of AI/ML in healthcare diagnostics was also common.^40,43,45,46,50,57,60^ Consumers expressed uncertainty about how to use AI/ML devices,^43^ about whether older consumers would be able to adapt to using AI/ML devices,^46^ and whether AI/ML devices would be accessible to people with low computer literacy or internet connectivity issues.^60^ There was also uncertainty about the immediate relevancy of AI/ML, with a perception that it would be something that ‘will happen, but not in the near future’.^45^ Of note, Canadian consumers who had uncertainty stated AI/ML was ‘at odds with cultural ways of knowing and healing’ and suggested ‘collaborating and partnering with community and cultural leaders may be helpful’ to ensure AI/ML was developed and implemented in appropriate ways.^40^

#### Trust

While distrust was a complex subtheme threaded throughout all the papers and across all the themes, it was also specifically discussed in relation to the development and implementation of AI/ML,^46,47,55,57^ and was greatly influenced by trust between consumers and doctors.^40,44–47,53,55–57,59,61^ There was distrust in the purpose of AI/ML, how it would be used, and by whom. Consumers expressly stated their distrust of insurance companies,^46,47^ pharmaceutical companies^46,47^ or other private companies^55^ that ‘were perceived to be driven by profit’.^47^ However, consumers ‘almost universally expressed a high level of trust in their physicians’^46^ and ‘if their physician determined the AI tool to be acceptable in terms of diagnostic accuracy and impact on health system processes, they would generally accept the tool as well’.^40^

#### Theme 2: Data use, privacy and governance

In this theme, three distinct but intertwined subthemes emerged from the literature. These were: (1) concerns about the types of data used to create AI/ML algorithms, (2) concerns about data use and its privacy and (3) concerns about data governance.

#### Data use

Consumers wanted control of what type of data is included and how this data is used in AI/ML algorithms. Consent processes for ‘certain information being taken and where that information is actually going to go’ were identified as necessary.^42^ For example, consumers stated ‘diagnosis, age and sex’^40^ data were acceptable for inclusion in AI/ML, but data such as residential address,^40^ mental health status^53^ and social media content^62^ were not. The quality and homogeneity of the data used in the AI/ML algorithms were also questioned,^42,54^ with concern ‘the data used to create the algorithm may not represent the vast majority of patients’.^54^ Furthermore, minority groups could be discriminated against as the ‘the (AI) system has been calibrated using data that isn’t particularly diverse and then the output it generates is biased because of that’.^42^ Within several studies,^42,50,53^ consumers expressed concern their data, and the subsequent AI/ML algorithms, could be used to discriminate against them, stating the concern ‘people at risk could be stigmatized and discriminated against’.^50^

#### Data privacy

Data security and privacy were key concerns of consumers on the use of AI/ML for healthcare. In fact, this issue was raised as a key concern in nearly all the papers.^40,42,45–48,50,53–55,58–62^ Consumers expressed particular concern on the security, and potential misuse, of their data.^42,46–48,50,54,55,59,60^ This included concern on how the data would be stored, stating there was the ‘potential for errors, breach of privacy, or loss of electronic information’^46^ and there was a perception that existing ‘data protection and security were … insufﬁcient’.^50^ Consumers expressed varying levels of distrust for current health system security measures,^42,47^ and the potential for ‘hacking’^44,47,60^ and ‘spying’^50^ on their personal health data. They had concerns about risks related to the ‘nefarious use of AI’,^55^ the potential for ‘ethical violations’^60^ and that ‘proﬁt-driven users of [health service] data might manipulate the data in ways that could burden or exploit patients, hinder medical decisions’.^48^ These consumer concerns underpinned a clear desire and need for security measures and safeguards to be in place.

#### Data governance

Effective data governance was identified as a prominent theme, with consumers emphasizing the requirement for close regulation surrounding the development and utilization of AI/ML tools.^42,46,48,50,54,55,61^ Suggestions of who had a role in governance included doctors,^46,48^ patients,^42,48^ government agenices,^42,46,48,54,55^ medical organizations,^42^ lawyers^48^ and technology companies.^55^ Consumers wanted opt-in consent procedures^42,61^ as well as the capacity to ‘accumulate and search’ their own health information.^49^ As illustrated in the following quote, consumers also suggested a broader shared responsibility among all parties for regulating the development of AI/ML tools:

… governing boards [should] include patient representatives along with experts, such as physicians, lawyers, and ethicists, who could ensure that patients are not burdened or exploited by the system.^48^

In comparison, consumers were clear about parties who they believed should not have a role in the governance development process, stating those who were in a position to profit were those that needed governance, including health services,^54^ pharmaceutical,^48^ marketing^46^ and insurance companies.^48^ This extended to the regulation of who could have access to use these tools, with some consumers stating the use of AI/ML tools should be restricted to healthcare providers.^46^ This was due to the consumer opinion that AI/ML in healthcare should only be used to improve the patient experience and health outcome, rather than for profit or marketing purposes.^46,48^ Consumers also suggested misuse of data should be penalized, as clearly illustrated in the following quote:

… for-proﬁt entities, especially pharmaceutical and insurance companies, must make clear how they intend to use the data, and if necessary, access should be restricted, fees should be charged for data use, and sanctions should be imposed if the data are not used accordingly…^48^

There were also suggestions on how the development and use of AI/ML tools should be funded,^49,50^ with consumers suggesting funding could come from ‘… taxpayers’ money or third-party funding from the private sector (manufacturers of drugs or medical devices)’.^50^

#### Theme 3: Consumers want to be engaged

Two distinct ways consumers want to be involved in the application of AI/ML in healthcare settings emerged from the data. These were (1) consumers want and need to know more about technology in healthcare and (2) consumers want and need input into how AI/ML is used in healthcare.

#### Consumers want to know more about healthcare technology

Consumers stated that patients need information and education about the use of AI/ML technology in healthcare.^40,42,43,45,46,50,51,59^ Patient education campaigns^40,45^ were identified as ‘necessary to increase patients’ acceptance of and input on how to best use AI systems in [healthcare]’.^45^ Clear information on how AI/ML tools work and why they are used was identified as imperative to avoid ‘confusion’.^43^ Key to this is clear communication between healthcare professionals and patients on how these tools are implemented as ‘patients’ level of knowledge of [machine learning in healthcare] may be rather limited’.^45^ Oral, in-person communication was described as preferrable,^46,59,61^ with the information being provided to the patient by their healthcare provider so patients have the opportunity to ‘ask questions and be reassured that their information would be anonymous and secure’*.*^46^ In-person explanations also allowed for ‘tailoring the discussion to consider each patient's level of understanding’*.*^46^ Ensuring consumers have ‘digital health literacy’ was seen as key to ‘strengthening [the] decision-making sovereignty’ of consumers and described as a ‘basic prerequisite for the implementation’ of AI/ML tools in healthcare.^42,50,61^

#### Consumers want input into how machine learning is used in healthcare

Consumers also want to know how AI/ML will be used in healthcare,^42,45^ including how the use of AI/ML will impact diagnostic testing and reporting procedures,^45^ the extent of their participation^47,51,61^ and how long their data will be included or their participation will be for.^47^ Consumers also wanted input into how information about AI/ML in healthcare is disseminated to consumers, stating it might not be clear to patients they are being ‘told that their data will be released to a [machine learning system]’ and how they are informed, as ‘some types of notiﬁcation, such as e-mail, may be overlooked’.^47^ Understanding how AI/ML will be used and having ‘trust in end users [use] of the data was critical for subjects to be comfortable with the concept’ of AI/ML in healthcare.^46^ Consumers were agreeable ‘data should be used to inform clinical decisions’,^48^ more broadly suggesting a willingness to integrate such tools into the diagnostic process,^48,57,61^ and AI/ML tools could be ‘both a dynamic and static diagnostic tool’.^55^ Taken together, this may further suggest consumers view these tools as a valuable aid to complement clinical decision making.

Closely linked to previous themes of concerns about data access to create AI/ML algorithms, consumers expressed a need for information about the clinical contexts in which AI/ML may be applied and who would use these tools. Some consumers made it clear there were contexts when the use of AI/ML tools would not be appropriate. For example, in one paper consumers did not like the concept of AI/ML for disease prediction stating that being informed they were ‘predisposed [or] a possible candidate for an illness is scary and concerning’.^58^ In another paper, consumers indicated AI/ML for suicide risk prediction was concerning and wanted to know how they ‘would be used, how and by whom’.^62^ Consumers were clearly uncomfortable with insurance and pharmaceutical companies using AI/ML tools for marketing or generating profit.^46,48,56^ The potential for developers to take shortcuts in the pursuit of profit was also discussed.^56^ However, evidence suggests consumers are comfortable with pharmaceutical companies using AI/ML tools if the purpose is to develop improved medical treatments^46,48^ and monitor adverse effects.^46^

#### Theme 4: Impact on healthcare delivery and access

Consumers expressed both positive and negative opinions on the potential impact of AI/ML tools on the patient experience. These opinions are captured within four clear subthemes: (1) the potential for AI/ML tools to improve the equitable access of healthcare for consumers; (2) the potential impacts on patient–doctor interactions, (3) the lack of clarity about healthcare responsibility and (4) potential impacts on physician training and skill development.

#### Potential improvements to equitable healthcare access

Consumers expressed their understanding of the many potential benefits of using AI/ML tools in healthcare. The most common potential benefit stated was faster diagnoses and, therefore, reductions in waiting times for treatment,^40,45,49–51^ quicker commencement of treatment,^40,43–45,48,50,53,55^ improved decision-making processes^42,44^ and a reduction in the strain on healthcare system.^49,54,55^ Consumers also identified faster diagnostic results could reduce the period of diagnostic uncertainty and the associated stress patients experience when waiting for test results.^40,41,55,56^ Consumers expressed the need for equitable access to the benefits of AI/ML^40,49,50^ regardless of geographic location of patients.^40,49^ Consumers also stated AI/ML could lead to reductions in healthcare costs^45,50^ and travel time^50,55^ for patients, and could result in increases in the volume of healthcare provided,^40^ leading to more patients being able to access diagnosis, care and treatment, including people in remote areas, the older persons and people with disabilities.^49,57^ In addition, consumers identified AI/ML has the potential to contribute to finding new treatments or cures,^46,48,53,59^ identifying simplified treatments,^49^ improving the efficacy and safety of treatments,^46,48,52,57^ and comparing treatments.^46^ These potential contributions and advancements facilitated by AI/ML to healthcare research and development has the capability to further improve healthcare quality and accessibility for patients.

#### Potential impacts on patient-doctor interactions

Across the literature, consumers expressed varying views on how AI/ML could potentially impact patient–doctor interactions. The main positive impact was the potential for improvement in communication between doctors and patients.^40,41,46,50,55^ This included potential for patients to feel more connected to their clinician,^41^ and the potential for patients to receive communication throughout their healthcare journey^40,46^ that is ‘independent of time and place’.^50^ The patient experience could also be improved through enhancements to clinical workflow, facilitated by the improved sharing of medical information between clinicians,^46,50^ including being able to ‘exchange health-related information in the care process across institutions and sectors’*.*^50^

Consumers were reported to have a favourable opinion of AI/ML diagnostic tools,^43,45,46,52,55,57^ perceiving these tools as ‘modern’^42,43,46^ and more accurate than current diagnostic technologies and techniques,^42,43,45,55,59^ as well as ‘superior’ or complementary to assessments done by healthcare practitioners alone.^43–45,52,53,57,59^ This is demonstrated by the following quote:

Patients perceived more accurate diagnosis … as the greatest strength of AI compared with human [diagnostic testing]. This perception was based on the ability of AI to draw on more data or experience than humans, to learn and evolve, and to share data.^55^

In addition, consumers also perceived AI/ML diagnostic tools as being less invasive compared to current methods,^43,55,57^ with the benefit of this being reduced patient anxiety.^49,55^

In contrast, a common concern of consumers was the potential for AI/ML tools to lead to a lack of human interaction with their healthcare providers.^40,42,45,46,49–51,55,58,60^ The preference for ‘in-person’^60^ and ‘social interaction’^55^ with their healthcare providers is clearly illustrated in the following excerpt:

… personal interaction when receiving information about the results of a scan is important to them; this human contact allows them to safely ask questions and to gain mutual understanding of the impact of results and reliability of ﬁndings.^45^

Across the literature, evidence demonstrates that consumers expressed reluctance towards the idea of AI/ML in replacing healthcare providers,^45,50–52,57,61^ stating ‘they did not want to lose the aspect of human interaction or to have [machine learning tools] replace doctors’^51^ and had concerns patients would be dehumanized,^45,50^ as clinical care became ‘depersonalized procedures in which patients become numbers’.^45^ Finally, there was also concern healthcare providers were increasingly distracted by technology during consultations, as illustrated in the following quote: ‘*…* they perceived that physicians focused too much on their computer and not enough on providing personal attention to their patients’.^46^ The sentiment that doctors may become overly reliant on such tools is echoed across other themes, such as the use of AI/ML to aid and inform clinical decisions, rather than replacing them, suggesting a potential overlap in consumers needing inclusion and governance concerns.

#### Lack of clarity about healthcare responsibility

Evidence from the literature demonstrates consumers need clarity about the use of AI/ML diagnostic tools. Consumers expressed concern that diagnostic technology could bias healthcare delivery.^46,47,50,53,62^ This included potential to ‘deny [insurance] coverage or treatment’ by insurance companies,^46^ and to stigmatize or discriminate against patients.^42,46,50^ Of concern was the perception ‘people at risk could be stigmatized and discriminated against in society because of individual predispositions or the prediction of disease events’.^50^ Furthermore, evidence demonstrates the use of healthcare technology may shift health responsibility from the doctor to the patient resulting in improved engagement of patients in their own healthcare.^40,41,50,55^ For example, consumers expressed they want to understand their results so ‘they can be engaged in their care’^40^ and – for consumers wearing a monitoring device – they can have ‘a sense of accountability that came with continuous monitoring … that this helped them cope … in a positive way’.^41^ However, consumers also expressed concern healthcare technology ‘disenfranchises people; it would take away their personal responsibility for their health’.^50^ Further to this, evidence shows consumers are aware there needs to be clear guidance on who is responsible for mistakes made by the AI/ML tools.^40,42,45,51,55,56^

In general, consumers noted healthcare professionals are ‘still critical to interpret the AI output’^40^ given that ‘AI can never replace humans’^51^ since ‘a human has the ability to … some form of consequential thinking’^61^ and ‘a computer is just a “giant calculator” … [so] humans will always be responsible’.^45^ In one paper, consumers discussed possible solutions for when the AI/ML tool and the healthcare professional had conflicting diagnoses.^55^ Consumers identified healthcare professionals ‘can be held accountable for their mistakes, and they wonder who can be held responsible for errors made by computers’*.*^45^ While this was explored in the literature, no clear answer was posed, with consumers suggesting the technology company, physician and/or the healthcare institution might be liable.^42,55,61^ Despite these uncertainties, consumers want to contribute to healthcare improvements and, in general, were content to have their data incorporated into AI/ML tools.^40,42,43,47,50,61^ This was most clearly stated in the following quote: ‘[consumers] are willing, and many eager, for their data to be shared to assist in the development of AI tools in health care’.^40^ However, there was acknowledgement that consumers’ intention is a balance between the ‘competing priorities of societal altruism versus respect for persons and protection of patients’^47^ and this needs to be incorporated into the development and implementation of AI/ML tools.

#### Potential impacts on physician training and skill

Throughout the literature, consumers discussed how AI/ML tools could support medical practitioners.^40,42,44–46,48,50,51,54,55,62^ There was general consensus these tools should support doctors and be integrated into healthcare delivery.^40,42,44,45,51,54,55^ Although, this integration was described as a potentially ‘radical change compared to how health is being practice now’*.*^53^ The tools were determined to be best used as a support tool to check diagnoses rather than a primary decision-making tool.^45,51,54^ AI/ML tools were also seen as a learning tool which could improve the education of healthcare professionals.^46^ Consumers want ‘their physicians to have the best tools available that are validated and trusted’,^40^ and believe these tools are ‘an opportunity for the medical process’^50^ which have the potential to improve medical decision-making processes. However, there was also concern that reliance on AI/ML tools could lead to a deskilling of the medical workforce.^42,54,55^ There was also concern the use of AI was ‘negating all the experience that [doctors] have … experience will tell them a lot of information that the machine can’t gather’.^42^

## Discussion

Our synthesized evidence demonstrates consumers’ understandings of AI/ML for medical diagnosis are complex. Consumers are both hesitant and supportive of the use of AI/ML in medical diagnosis with their views influenced by the perceived trustworthiness of their healthcare providers who use these AI/ML tools. Their main emergent concerns were the privacy and governance of the data used in AI/ML tools and who was responsible for ensuring data security. Consumers were also concerned about the potential impact of AI/ML tools on healthcare delivery and access, including the potential to depersonalize their interactions with their healthcare providers and the shifting of healthcare responsibility from the healthcare providers to consumers. However, positively, consumers recognized the potential for AI/ML tools to improve diagnostic accuracy, efficiency, and access, and are keen to be engaged in the development and implementation processes associated with AI/ML diagnostic tools.

The importance of including consumers in healthcare design is increasingly apparent. As the end-users of these healthcare services, consumers play a crucial role in ensuring these tools are designed, implemented and evaluated to meet their specific healthcare needs and preferences.^63^ Including consumers can improve the quality of healthcare,^64^ ensure healthcare remains person centred,^65^ improve efficiency,^63^ and build trust.^63^ Consumers are increasingly included on advisory boards, providing feedback on existing services as well as on the design and testing of new healthcare products.^63^ As a result, it is logical to also engage consumers in the design, implementation and evaluation of healthcare-related AI/ML tools.

Evidence of consumer acceptance of AI/ML in healthcare is still fairly limited but has been demonstrated for a variety of health technologies, including AI-based symptom checkers (AISC)^66,67^ and wearable technologies.^68,69^ Thus far, evidence suggests the users of these technologies are younger^66^ and are socially influenced on whether to use these technologies.^68^ Users of AISC find the tools useful for diagnosis despite ongoing concerns about accuracy.^67^ However, most current literature evaluating consumer engagement with AISC continues to focus on the usability and functionality of the AISC rather than the users’ experience,^66^ and most studies have sought feedback from consumers in controlled experimental settings rather than in real-world scenarios, lacking ecological validation.^66,67^ While the importance of person-centred care is often emphasized,^70^ evidence shows it is not centralized or foundational in design processes.^66^

A major concern of consumers evident from our synthesis was data privacy and security. Healthcare services are now reliant on technology to function effectively. This includes medical records housed in electronic systems, clinicians engaging in providing healthcare via telehealth platforms and electronic transfer of data between healthcare service providers and with government for healthcare system planning and budgeting. As a result, there are huge amounts of confidential medical data stored in and shared across electronic platforms,^71^ and while there are benefits of digital health data storage, such as convenience, scalability and analytics,^72–74^ these data are often stored with internet connectivity putting the data at risk of cybersecurity breaches.^71^ As a result, a key challenge of using digital health data is data security and ensuring consumer confidence in data security measures. Breaches in health data security can have severe impacts for consumers’ personal lives, including bullying, increased private insurance costs and termination of employment due to previous medical history.^71^ Furthermore, high-profile cases of health data security breaches, such as the Australian health insurance provider Medibank health data breach,^75^ or the controversial use of health data, such as the use of NHS data by Google's DeepMind app,^76^ have highlighted the ongoing challenges in the use and storage of electronic health data, further compounding issues of confidence and trust in data privacy and security among consumers.

Protecting health data from unauthorized access is complex, with proposed solutions for achieving data privacy including the use of blockchain;^77^ however this in itself creates confidentiality problems as the data are then stored within the blockchain network.^78^ As a result, it is vital to ensure data storage is compliant with privacy laws and regulations, such as the Health Insurance Portability and Accountability Act (HIPAA) legislation in the USA or Regulation (EU) 2016/679 of the European Union.^78–80^ However, even these privacy protections have limitations for data stored outside of healthcare systems,^80^ such as when consumers use health apps, AISC and wearable technologies. Healthcare consumers and organizations need clear policies on how, where and for what duration health data will be stored, who has access to the data and for what purposes, and who is responsible for enforcing the policies.

## Strengths and limitations

The clear strength of this paper is the rigorous and systematic JBI methodology followed by the authors to identify the key themes from the existing body of literature on this topic. The methodology has been previously well documented and is a robust method for conducting qualitative systematic reviews. However, as with any systematic review, there are potential limitations which need to be considered. Firstly, we acknowledge the included papers used various study designs, which means data were extracted from studies not specifically designed to collect qualitative data. This may have impacted on the quality of the data collected and reported in these studies, particularly if the data were collected by researchers unfamiliar with qualitative research methods. This was further highlighted by the variable quality of the included studies, particularly with the data collection and analysis methods poorly reported in several of the papers. The number of papers identified for inclusion in the systematic review was also small which may impact on the generalizability of the findings. However, the small number of identified papers, and a lack of grey literature, is probably a reflection on the emerging nature of this research field.

## Conclusions

The meta-synthesis reported herein has clearly demonstrated the complex nature of consumer opinions regarding the use of AI/ML in healthcare diagnostic tools. Most surprising is that despite the ever-increasing number of AI/ML tools, there is little research into consumer perspectives on the use of these tools for diagnostic investigations. Despite limited evidence, and although consumers express some hesitancy regarding the use of AI/ML tools, they also recognize their potential value in healthcare. Notably, consumers are integral to the acceptability and integration of AI/ML tools into healthcare systems and, as such, there must be widespread inclusion of consumer perspectives in discussions on the design, development and implementation of AI/ML diagnostic tools.

## Supplemental Material

sj-docx-1-dhj-10.1177_20552076241288631 - Supplemental material for Consumer opinion on the use of machine learning in healthcare settings: A qualitative systematic review Supplemental material, sj-docx-1-dhj-10.1177_20552076241288631 for Consumer opinion on the use of machine learning in healthcare settings: A qualitative systematic review by Jacqueline H Stephens, Celine Northcott, Brianna F Poirier and Trent Lewis in DIGITAL HEALTH
